# The Role of Extracellular Vesicles in the Pathogenesis and Treatment of Rheumatoid Arthritis and Osteoarthritis

**DOI:** 10.3390/cells12232716

**Published:** 2023-11-27

**Authors:** Estera Bakinowska, Kajetan Kiełbowski, Andrzej Pawlik

**Affiliations:** Department of Physiology, Pomeranian Medical University, 70-111 Szczecin, Poland; esterabakinowska@gmail.com (E.B.); kajetan.kielbowski@onet.pl (K.K.)

**Keywords:** extracellular vesicles, exosomes, rheumatoid arthritis, osteoarthritis, mesenchymal stem cells

## Abstract

Cells can communicate with each other through extracellular vesicles (EVs), which are membrane-bound structures that transport proteins, lipids and nucleic acids. These structures have been found to mediate cellular differentiation and proliferation apoptosis, as well as inflammatory responses and senescence, among others. The cargo of these vesicles may include immunomodulatory molecules, which can then contribute to the pathogenesis of various diseases. By contrast, EVs secreted by mesenchymal stem cells (MSCs) have shown important immunosuppressive and regenerative properties. Moreover, EVs can be modified and used as drug carriers to precisely deliver therapeutic agents. In this review, we aim to summarize the current evidence on the roles of EVs in the progression and treatment of rheumatoid arthritis (RA) and osteoarthritis (OA), which are important and prevalent joint diseases with a significant global burden.

## 1. Introduction

Extracellular vesicles (EVs) are membrane-bound structures secreted by donor cells which can carry proteins, lipids and nucleic acids. Depending on their biogenesis and size, several types of EVs have been characterized, including exosomes, microvesicles, apoptotic bodies (ABs) and oncosomes. Exosomes originate from intraluminal vesicles after the fusion of multivesicular endosomes (MVEs) with the plasma membrane, while microvesicles are formed from the plasma membrane and contain cytoplasmic content. ABs are released by dying cells, and oncosomes are the products of neoplastic cells [1]. These structures can transport active cargo into surrounding or distant cells, which would alter their functions. Recent studies have uncovered that EVs take part in multiple processes, such as cellular communication, immunomodulation or even tumor progression [2]. Immunomodulatory properties of EVs are of particular interest, as they can regulate the progression of inflammatory or neoplastic diseases. For instance, EVs can regulate the polarization of macrophages and thus contribute to the progression of atherosclerosis or lung cancer [3,4]. Importantly, EV-based nanoparticles can be used for treatment purposes to deliver the required treatment agent directly to disease-related cells [5]. In this review, we will try to present the current evidence regarding the role of EVs in the pathogenesis and treatment of rheumatoid arthritis (RA) and osteoarthritis (OA).

## 2. Extracellular Vesicles in the Pathogenesis of Rheumatoid Arthritis

### 2.1. Extracellular Vesicles and Rheumatoid Arthritis

Rheumatoid arthritis is one of the most frequent inflammatory diseases, and causes structural and functional joint impairment. Nevertheless, the disease is also characterized by extraarticular manifestations, such as vasculitis, pulmonary involvement or rheumatoid nodules. Synovial inflammation, bone erosion and cartilage damage are key elements of RA pathogenesis. Multiple cells are involved in the progression of RA, including fibroblast-like synoviocytes (FLSs), macrophages, osteoclasts, neutrophils and T cells, among others [6]. These cells communicate through cytokines, and interactions between them impact cellular phenotypes and create a pro-inflammatory environment. Importantly, recent studies highlighted the vital role of EVs in these interactions. As previously mentioned, EVs can contain proteins, lipids or nucleic acids. The latter often indicate non-coding RNA (ncRNA), such as micro-RNA (miRNA), long non-coding RNA (lncRNA) or circular RNA (circRNA). Briefly, these molecules regulate gene expression. For instance, miRNAs can suppress translation while lncRNA and circRNA can act as competing endogenous RNA (ceRNA) or sponges that sequestrate miRNAs.

To begin with, RA patients have elevated levels of EVs compared to healthy controls [7]. Furthermore, altered cargo of EVs in rheumatoid patients has been detected, which may be useful in the diagnosis process [8]. Plasma EVs may contain a rheumatoid factor (RF), an autoantibody which is used in the diagnosis of RA. The presence of these structures could become a novel biomarker of disease severity. Arntz et al. demonstrated that patients with IgM-RF-positive plasma EVs had significantly higher DAS28, ESR, and VAS scores, as well as elevated C-reactive protein (CRP) concentrations, compared to seropositive patients without the expression of RF on EVs [9]. Furthermore, strong correlations between the level of RF and the number of B- and T-cell-derived microvesicles were observed [10]. Importantly, Stojanovic and colleagues showed significant correlations between plasma EVs and CRP levels, together with coagulation parameters in female RA patients. The authors observed significantly more EVs expressing a tissue factor (TF) in the RA cohort than in healthy controls [11]. A previous study demonstrated a strong immunohistochemical expression of TF in the rheumatoid synovium and suggested that it could play a role in RA progression [12]. EVs’ express markers represented their parent cells, as well as EV-specific markers. The membrane of the vesicles contains tetraspanins, which are proteins involved in important cellular processes, such as cell adhesion, membrane fusion and protein trafficking, among others. It has been suggested that EVs could be identified using these markers. For instance, membranes of the exosomes contain CD9, CD37, CD63 and CD81 [13]. Indeed, the expression of single CD9 and CD81 on the surface of small EVs isolated from blood plasma was elevated in RA patients [14]. An early study by Nakagawa et al. demonstrated that the introduction of CD81 siRNA to the joints of collagen-induced arthritis (CIA) rats reduces clinical symptoms and joint damage [15]. The intra-articular administration of an RNA vector expressing anti-CD81 antibodies also showed benefits in CIA-rats [16]. Interestingly, the monitoring of tetraspanins could become useful in evaluating the response to treatment. Rydland et al. showed that responders to methotrexate demonstrate higher CD81 expression that non-responders [14].

### 2.2. Autoimmunity

The pathogenesis of RA is associated with the formation of autoantibodies, such as RF, anti-citrullinated protein antibodies (ACPA), and anti-carbamylated protein antibodies. RF targets the Fc region of immunoglobulin G (IgG), while ACPA recognizes proteins that undergo post-translational citrullination. Collagen, vimentin and fibrinogen are some of the antigens undergoing this modification [17]. Studies have shown that the presence of ACPA is correlated with severe disease, and that these antibodies can contribute to the progression of the disease by promoting inflammatory responses [18].

EVs have been suggested to take part in the pathogenesis of autoimmune diseases. They are opsonized by CRP, which could drive the production of anti-CRP autoantibodies present in systemic lupus erythematosus (SLE) [19]. Moreover, EVs isolated from the islet beta cells have also been found to contain diabetic autoantigens [20]. Synovial exosomes derived from patients with articular diseases (OA, RA and reactive arthritis) have been found to contain citrullinated proteins [21]. Recently, a study by Ucci et al. suggested that extracellular microvesicles present citrullinated antigens on the surface of vesicles. The higher expression of citrullinated antigens has been detected in RA-microvesicles compared with those of healthy donors. In addition, the presence of citrullinated antigens was positively correlated with disease activity [22]. Importantly, EVs carry the peptide-major histocompatibility complex (MHC) and present antigens to T cells [23]. Furthermore, they can take part in the formation of immune complexes (ICs). ICs with citrullinated peptides induce inflammatory responses by stimulating the production of the tumor necrosis factor (TNF) by macrophages [24]. TNF plays a major role in the pathogenesis of RA, as this cytokine is involved in inflammatory responses and bone resorption [25]. Cloutier and collaborators found that rheumatoid synovial fluid contains ICs associated with microparticles (MPs). The latter structures were observed to contain citrullinated epitopes and can display vimentin and fibrinogen. The authors found that MP-ICs can promote the secretion of leukotriens by neutrophils [26]. Furthermore, such structures were found to induce pro-inflammatory responses in monocytes in an in vitro study. Villar-Vesga and colleagues demonstrated that the protein expression of interleukin (IL)-1β, IL-6 and TNF-α was greater in stimulated monocytes derived from healthy individuals, which could be related to the state of tolerance of rheumatoid cells [27]. However, another study by Burbano et al. detected elevations of pro-inflammatory cytokines (IL-1β, IL-6 and TNF-α) in supernatants of mononuclear phagocytes in the presence of EVs, and EV-ICs form seropositive patients [28]. Furthermore, a study investigating MPs with ICs from patients with RA and SLE showed that these structures can promote the expression of adhesion molecules by endothelial cells [29]. Thus, RA-EVs might be the source of autoantigens, driving the autoimmunity. In addition, ICs that form with the EVs can promote inflammation in immune cells, which can also contribute to the chronic inflammatory conditions.

### 2.3. Inflammation

Chronic inflammation is a major hallmark of RA. The pathological environment of the affected joint involves multiple cells, which interact with each other to stimulate the secretion of pro-inflammatory cytokines, chemokines and proteases. EVs can propagate the inflammation, as these structures may contain or express inflammatory molecules. For instance, EVs can carry TNF-α on their membrane and stimulate the nuclear factor kappa B (NF-κB) pathway, which is involved in the progression of inflammatory diseases [30]. In RA, MPs were found to express TNF-α on their surface. MPs derived from endothelial cells demonstrated a greater abundance of TNF-α in RA than those derived from healthy controls. Importantly, positive correlations between the percentage of MP-associated TNF and clinical rheumatoid parameters have been observed. A treatment with etanercept (TNF inhibitor), but not with classical disease-modifying anti-rheumatic drugs (DMARDs), reduced the surface expression of TNF [31]. Furthermore, the membrane-bound form of TNF-α was found on the vesicles derived from RA-FLSs. These cells play a major role in the progression of RA. RA-FLSs have a tumor-like appearance due to similarities with malignant cells, such as a high expression of oncogenes and abnormal growth, as well as elevated telomerase activity or secretion of chemokines to attract immune cells and promote inflammation or angiogenesis [32]. An early study by Zhang et al. demonstrated that RA-FLS-derived exosomes with TNF-α-stimulated NF-κB and thus, promoted the secretion of the matrix metalloproteinase 1 (MMP1) in RA and OA synovial fibroblasts [33]. Therefore, RA-FLS-derived exosomes may stimulate inflammatory responses in other FLSs and induce tissue degeneration.

Additionally, despite the ability to directly promote inflammation by transporting TNF-α, synovial cells react to inflammatory stimuli and thus, secrete vesicles with particular cargo that can further regulate inflammation. For instance, the treatment of RA-FLS with TNF-α promotes the secretion of exosomes with miR-155-5p, miR-1307-3p, miR-323a-5p and miR-146a-5p [34]. MiR-155 has been previously associated with the pathogenesis of RA. Studies have found that miR-155 promotes inflammatory responses in macrophages and suppresses the anti-inflammatory polarization. Moreover, it plays a role in the antibody production in RA [35]. In the study by Takamura et al., the authors predicted that miR-323a-5p targets CD6, a T cell signaling attenuator. Consequently, RA-FLS-derived exosomes containing miR-323a-5p could enhance T cell responses [34,36]. Moreover, MPs derived from synovial fluid can promote inflammatory responses in FLSs. These structures promote the release of IL-6 and IL-8, major mediators of inflammation and chemotaxis, respectively [37]. Exosomes secreted by RA-FLS also promote macrophage migration [38]. In the study by Nakamachi et al., the authors demonstrated that the stimulation of macrophage migration could be mediated by pentraxin 3 (PTX3). Silencing PTX3 resulted in statistically significant reduction in the number of migrated cells [38]. PTX3 is an inflammatory marker elevated in the serum of RA patients compared to healthy controls [39,40]. Plasma PTX3 expression has been positively correlated with the concentration of MMP-3, CRP, and radiographic joint damage. Therefore, these studies suggest that PTX3 contributes to the progression of RA [41].

Moreover, EVs secreted by synovial fibroblasts regulate T cell differentiation. For instance, exosomes containing miR-424 promote the differentiation of Th17 cells [42]. The presence of these cells, together with Th17-related cytokines, have been correlated with disease activity. Importantly, Th17 cells can promote inflammation by activating FLSs and macrophages [43]. Th17 cells are an important source of IL-17, a cytokine implicated in RA progression. It has been demonstrated that IL-17 induces mitochondrial dysfunction in RA-FLSs and inhibits apoptosis. Therefore, Th17 cells and IL-17 could contribute to the tumor-like features of rheumatoid FLSs [44]. Overall, cells use EVs to promote inflammation through various methods. These structures can express inflammatory molecules that directly induce inflammatory responses. Furthermore, activated cells secrete vesicles to induce cellular migration or differentiation.

### 2.4. Bone Degeneration

The chronic inflammation of RA is associated with an imbalance between bone formation and loss. This impairment is caused by the stimulation of osteoclasts through the binding of the receptor activator of NF-κB ligand (RANKL) to its receptor RANK, which is expressed on the precursor of osteoclasts [45]. EVs seem to be broadly used to regulate the differentiation of cells involved in the bone homeostasis. Interestingly, Uenaka et al. suggested that even osteoblasts can secrete vesicles that promote RANKL expression and osteoclastogenesis [46].

In RA, EVs seem to take part in the bone degradation by the modification of osteoclast/osteoblast balance. Exosomes isolated from RA-FLS co-cultured with osteoblasts suppress their differentiation [47]. An in vitro experiment showed that FLS stimulated with TNF secrete exosomal miR-221-3p, which can suppress osteoblast differentiation through the targeting of Dickkopf2 (Dkk2) [48]. Dkk2 has been previously found to mediate osteoblast differentiation. Despite being a Wnt-signaling antagonist, the regulatory role in osteoblast differentiation could be Wnt-independent [49]. Nonetheless, the role of Dkk2 in osteogenesis might be more complex, as Zhou et al. demonstrated that the knockdown of Dkk2 enhances osteoblast proliferation [50]. MiR-221-3p is upregulated in RA-FLS compared to healthy controls. Its expression is positively correlated with the urokinase-type plasminogen activator receptor (uPAR), a molecule which has been associated with RA-related inflammation and joint damage [51,52]. Moreover, miR-221-3p takes part in the regulation of macrophage polarization and promotes the secretion of pro-inflammatory molecules from M2 macrophages through the JAK3/STAT3 pathway [53]. In addition, ncRNA in EVs can determine the differentiation of osteoclasts. For instance, an elevated expression of miR-574-5p is detected in RA-FLS-derived small EVs from ACPA-positive patients. Mir-574-5p stimulates toll-like receptor (TLR) 7/8 and promotes osteoclast maturation [54]. In addition, EVs derived from the previously mentioned Th17 cells can also contribute to the RA progression. Cigarette smoke-enriched medium or aryl hydrocarbon receptor (AhR) agonist promote the expression of miR-132 in Th17-derived EVs, which then promotes osteoclastogenesis by targeting cyclooxygenase-2 (COX-2) [55]. Despite regulating osteogenesis, microvesicles secreted by RA-FLS contain enzymes which can degrade the extracellular matrix (ECM). Precisely, Lo Cicero and colleagues demonstrated that these structures contain proteases able to degrade aggrecan, which was reversed by the tissue inhibitor of metalloproteinases (TIMP)-3 [56].

### 2.5. Angiogenesis

**Figure 1:**
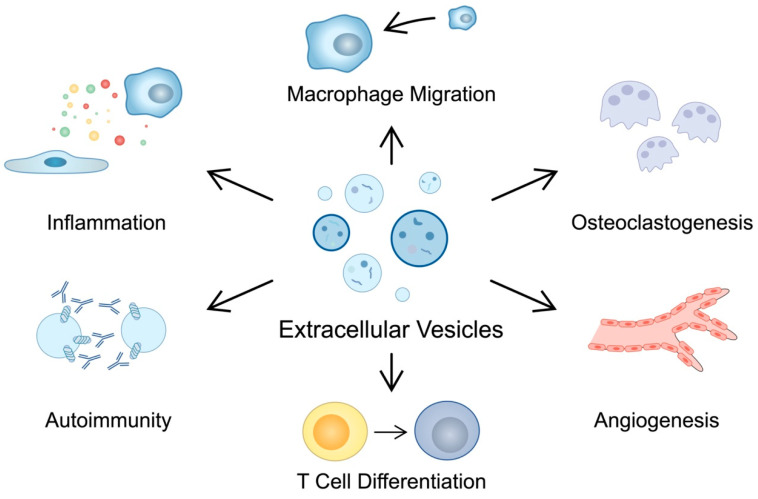
Summary of the mechanisms involving EVs that take part in the pathogenesis of RA. These structures have been found to contribute to autoimmune reactions, inflammatory responses, macrophage migration, bone impairment, angiogenesis and T cell differentiation.

Physiologically, angiogenesis is an important mechanism taking part in tissue repair or wound healing. Nevertheless, in chronic inflammatory conditions present in RA, angiogenesis can facilitate the infiltration of immune cells to affected articular tissues. One of the key regulators of angiogenesis is the vascular endothelial growth factor (VEGF), a molecule which has been implicated in the progression of RA [57]. Rheumatoid fibroblasts produce the inhibitor of DNA binding 1 (id1), the majority of which is located in exosomes. The transforming growth factor (TGF) is a strong inducer of id1, which has pro-angiogenic features, thus contributing to the progression of RA [58]. Serum ID-1 demonstrates positive correlations with clinical and laboratory markers, including ESR, DAS28, MMP-3 and CRP. Importantly, ID-1 can be citrullinated and thus can contribute to autoimmunity in RA [59]. Moreover, RA-FLS interact with endothelial cells to promote angiogenesis. EVs derived from RA-FLS stimulate migration of endothelial cells and tube formation. For instance, EVs derived from RA-FLS and lipopolysaccharide (LPS)-stimulated RA-FLS contain miR-1972 which then targets p53 and promotes mTOR phosphorylation, which acts in favor of angiogenesis [60]. Mechanisms involved in the pathogenesis of RA are summarized in Table 1 and Figure 1.

## 3. The Role of Extracellular Vesicles in Suppressing Rheumatoid Arthritis

### 3.1. Mesenchymal Stem Cells and Rheumatoid Arthritis

In the previous section, we discussed the pro-inflammatory roles of EVs and their contribution to the pathogenesis od RA. Nevertheless, studies showed that EVs can also suppress the disease progression. Interestingly, these structures are being used to facilitate the immunomodulatory properties of mesenchymal stem cells (MSCs). They are progenitor cells found in a variety of tissues such as adipose tissue (AD-MSCs), placenta, bone marrow (BM-MSC), human umbilical cord (UC-MSC) and gingiva (GMSCs) [61]. MSCs affect other cells by their paracrine properties, which can be mediated by the cargo of EVs. The immunosuppressive and regenerative properties of MSC-derived EVs have led to investigations on whether these structures can inhibit the progression of inflammatory diseases. Furthermore, a number of studies have investigated the genetic modifications of MSCs, which then secrete EVS with a desired cargo.

To begin with, studies have shown that the application of MSC reduces the disease biomarkers, as well as inflammation in the animal arthritis models [62,63]. The promising results of the preclinical studies led to the investigation of MSCs in the therapy of RA in clinical trials [64,65]. Furthermore, BM-MSCs show modulatory effects on RA-derived T cells, as they decrease the production of pro-inflammatory cytokines, including TNF-α, IL-17, and IL-6, among others [66]. Nonetheless, the use of MSCs is associated with certain limitations. High viability, resistance to apoptosis and the ability to proliferate could be associated with the risk of tumorigenesis. Moreover, differentiated stem cells might have a higher immunogenicity. The repeated application of MSCs could be associated with the generation of allo-antibodies [67]. The use of MSC-EVs is an interesting approach for a cell-free therapy. Importantly, the beneficial role of MSCs in RA can be conferred by EVs [68].

### 3.2. Mesenchymal Stem-Cell-Derived Extracellular Vesicles and Fibroblast-like Synoviocytes

FLSs play a key role in the progression of RA. As previously mentioned, they have an invasive character (“tumor-like appearance”) and secrete proteases and pro-inflammatory cytokines. Furthermore, these cells demonstrate enhanced survival and proliferation [69]. As a result, regulating pathological features of RA-FLSs could become clinically beneficial. Several studies have shown that MSC-derived EVs can suppress the invasive properties of RA-FLSs. Firstly, in a study by Bruckner et al., the authors showed that GMSCs and their exosomes inhibit the invasiveness of RA-FLSs by suppressing the formation of a pannus-like structure in vivo [70]. In another study, Wu and colleagues demonstrated that BM-MSC-derived EVs containing miR-34a reduce proliferation and induce apoptosis in RA-FLSs. Moreover, in an in vivo experiment, the introduction of EVs inhibited inflammation by suppressing IL-6, IL-8 and TNF-α. In addition, the disease progressed significantly faster in the cohort treated with EVs containing an miR-34a inhibitor. MiR-34a inhibits cyclin I which activates the ATM/ATR/p53 pathway [71]. Meng et al. prepared exosomes derived from human MSCs that overexpressed miR-124a. The authors co-incubated MSC-derived EVs and EVs overexpressing miR-124a with RA-FLSs, and observed that the proliferation was suppressed in both cohorts, but the inhibition was greater in the group with modified vesicles [72]. MiR-124a has been previously found to be downregulated in rheumatoid synoviocytes, as compared to OA cells. Moreover, an early study demonstrated that miR-124a can suppress proliferation through the interaction with cyclin-dependent kinases (CDKs) [73]. Due to the downregulation of miR-124a in plasma, peripheral blood mononuclear cells (PBMC) and synovial fluid, as well as negative correlations with clinical parameters, it has been proposed that miR-124a could be used in the diagnosis process of RA [74]. In addition, exosomes derived from chondrogenic bone marrow stem cells contain miR-205-5p, which targets MDM2 and reduces inflammatory responses in FLSs. In an in vivo study, the application of exo-miR-205-5p suppressed pro-inflammatory cytokines [75]. MiR-320a is another molecule downregulated in the synovial tissues of RA patients. It mediates the proliferation and apoptosis pathways by interacting with ERK [76]. Importantly, MSCs secrete exosomes with a high expression of miR-320a. Experiments using MSCs treated with miR-320a mimics demonstrated that exosomes containing this microRNA could suppress the invasion of RA-FLSs, through the interaction of miR-320a with CXCL9, a member of the chemokine family upregulated in RA and positively correlated with DAS-28 [77,78,79]. However, the role of certain molecules may depend on the cellular context. A study by Qiu et al. demonstrated that miR-150-5p notably reduced levels of the suppressor of cytokine signaling 1 (SOCS1) and promoted the growth of RA-FLSs, which suggested its potential role as a therapeutic target in RA [80]. By contrast, Chen and colleagues revealed that the expression of miR-150-5p is reduced in the serum and synovial tissue in patients with RA compared to those with OA. The authors transfected MSCs with a miR-150-5p plasmid and subsequently isolated MSC-derived vesicles overexpressing this RNA molecule (Exo-150). These vesicles inhibited the invasion and migration in RA-FLSs. In addition, investigated structures suppressed tube formation by endothelial cells. After examining the mechanism of action, the authors demonstrated that miR-150-5p targeted VEGF and MMP-14 in FLSs. Exo-150 reduced the IL-1β-induced expression of VEGF as well as that of MMP-14 [81]. Furthermore, the expression of miR-140-3p is decreased in rheumatoid tissues compared to normal synovium. The molecule targets SIRT3, a protein which mediates apoptosis and increases cell viability. As a result, the overexpression of miR-140-3p could play a beneficial role in RA [82]. The transfection of UC-MSC-derived exosomes with miR-140-3p resulted in the suppression of proliferation and accelerated apoptosis of RA-FLSs through the inhibition of SGK1. MiR-140-3p overexpressing exosomes also demonstrated important activity in vivo, as they significantly reduced the severity score in RA rat models [83]. MiR-21 plays a role in the pathogenesis of malignancies by mediating the expression of tumor suppressors [84,85]. However, the inhibition of miR-21 in BM-MSC-derived EVs resulted in an elevation of pro-inflammatory cytokines expressed by mouse FLSs. In this study, miR-21 acted via targeting of the TET1/KLF4 axis. This promoted the proliferation of FLSs but inhibited the secretion of inflammatory cytokines. The use of an miR-21 mimic in EVs resulted in decreased inflammation markers, together with clinical ad histological scores in collagen-induced arthritis (CIA) animal models [86]. Nonetheless, the role of miR-21 in the pathogenesis of autoimmune diseases is less known. In early rheumatoid and psoriatic arthritis, miR-21-5p was overexpressed compared to that in healthy controls [87]. On the contrary, the study by Dong et al. showed that the expression of miR-21 is lower in RA patients than in healthy controls. Moreover, the level of miR-21 has been correlated with the Th17/Treg ratio [88].

Limited studies have evaluated encapsulated lncRNAs or circRNAs and the use of their properties as sponges of miRNAs. For instance, the expression of miR-143-3p is upregulated in RA patients. The inhibition of miR-143-3p promoted apoptosis and inhibited the expression of pro-inflammatory mediators in human RA MH7A synovial cells. Therefore, it was suggested that miR-143-3p could become a therapeutic target [89]. Subsequently, a study by Su et al. investigated the transfection of MSC-secreting exosomes with the HAND2-AS1 plasmid, a lncRNA which sponges miR-143-3p. The sssimilation of these structures by RA-FLSs was associated with inhibited inflammation. In this study, miR-143-3p was found to target TNFAIP3, an NF-κB inhibitor [90]. However, conflicting results regarding the role of miR-143-3p in RA have been published. Shen and colleagues demonstrated that the expression of the abovementioned miRNA is elevated in CD4+ cells in moderate RA but decreased in severe disease, as compared to healthy controls. Moreover, the authors observed that miR-143-3p mimics reduced joint inflammatory scores [91]. Furthermore, MSCs-EVs were mixed with curcumin, a natural herbal medicine, which showed anti-proliferative, anti-inflammatory and pro-apoptotic properties in synovial cells [92]. Overall, the ability of RA-FLSs to accumulate MSC-derived EVs represent an exciting method to precisely deliver therapeutic agents that can modulate the activity of RA-FLSs. The summarized studies demonstrate beneficial effects of molecules inhibiting RA-FLSs’ invasiveness and proliferation, as well as those that promote apoptosis. Targeting RA-FLSs is being investigated as potential method of RA treatment [93].

### 3.3. Mesenchymal Stem-Cell-Derived Extracellular Vesicles and T Cells

In RA, multiple T cell subtypes with the potential to contribute to RA progression have been identified [94]. Moreover, an imbalance between T cells has been observed, such as the widely studied Th17/Treg ratio [95,96]. Studies have demonstrated that EVs can modulate T cell responses as well as their differentiation. The differentiation of T cells depends on the expression of specific transcription factors, which promote gene patterns typical for the final subtype. For instance, t-bet is considered as a regulator of Th1 cells, while the retinoid acid-related orphan receptor (ROR)γt is associated with the Th17 differentiation [97]. Recently, Bolandi et al. demonstrated that MSC-derived exosomes containing miR-29b can modulate the expression of several major T cell transcription factors, including RORγt [98]. Small EVs derived from UC-MSCs inhibit synovial hyperplasia and suppress arthritis in animal models. Importantly, these structures affect T cell differentiation, as they reduce the proportion of Th17 and promote the Treg cells population [99]. Exosomes derived from GMSCs also reduce the percentage of Th17 cells and decrease IL-17 levels. In addition, GMSC-derived exosomes suppressed arthritis in an in vivo experiment [100]. Intriguingly, UC-MSC-EVs primed with pro-inflammatory mediators showed upregulation of FoxP3 in peripheral blood mononuclear cells [101]. As previously mentioned, encapsulated miR-21 could alleviate RA [86]. Moreover, it could be associated with T cell differentiation, due to its interaction with STAT3. This inhibits the transformation of Tregs into the cells secreting IL-17 [102]. Similarly, another study demonstrated that Maresin1, a macrophage-derived mediator, suppressed Th17 and enhanced the Treg differentiation by miR-21 overexpression [103].

### 3.4. Mesenchymal Stem-Cell-Derived Extracellular Vesicles and Macrophages

Macrophages take part in RA progression, as they are the major source of the pro-inflammatory TNF-α. These cells can present different phenotypes, which is associated with their role in inflammation. M1 polarization is associated with pro-inflammatory responses that take part in the pathogenesis of RA [104]. Thus, targeted therapies able to modify macrophage polarization are of great interest. The use of MSCs could represent such a strategy. IL-1β-stimulated UC-MSCs promote the anti-inflammatory M2 phenotype and induce apoptosis of the M1 cells [105]. Since MSCs secrete EVs that show immunomodulatory properties resembling their parental cells, these structures could modify the macrophage phenotypes as well. Indeed, as demonstrated by Choi and colleagues, priming AD-MSCs with RA disease serum resulted in a higher production of exosomes, which contained elevated TGF-β1 levels and promoted the M2 macrophage phenotype in the **CIA** models [106]. IL-4 is a cytokine that promotes the M2 polarization. The use of IL-4 with MSCs has shown benefits in RA animal models [62]. Interestingly, a modification of small EVs-producing cells to secrete vesicles containing IL-4 promoted the polarization of macrophage towards M2 and achieved beneficial effects in animal models [107]. Therefore, the use of EVs to control macrophage polarization seems to be a potential future strategy to reduce inflammation.

### 3.5. Extracellular Vesicles and Programmed Cell Death Pathways

Programmed cell death protein 1 (PD-1) is a negative regulator of T cell responses. It binds to two ligands (PD-L1 and PD-L2), and its activation is associated with tolerance. In RA, it has been suggested that the PD-1/PD-L1 axis may be dysfunctional, and the ligand may not be available in the inflamed synovium [108]. Interestingly, the intra-articular delivery of PD-L1 vectors decreased the histopathological score, as well as pro-inflammatory cytokine concentrations in CIA mice [109]. Hypothetically, PD-L1 could be delivered with the use of EVs. MSC-derived EVs modified to express PD-L1 were found to induce beneficial effects in the model of ulcerative colitis and psoriasis [110]. In addition, He and colleagues demonstrated that EVs with PD-L1 could reduce the proportion of Th17 cells and promote Tregs in colitis rat models [111]. Therefore, the results of studies evaluating PD-L1-EVs suggest that similar structures could be investigated in the treatment of RA.

## 4. The Role of Extracellular Vesicles in the Pathogenesis of Osteoarthritis

### 4.1. Osteoarthritis and Extracellular Vesicles

Osteoarthritis is a whole joint disease with a significant prevalence and burden. The pathogenesis of OA involves changes in the synovium, articular cartilage, subchondral bone, muscles and ligaments. Metabolic, mechanical and inflammatory factors contribute to structural joint impairment [112]. Studies demonstrated the presence of inflammatory cells in OA tissues and suggested their important roles in the progression of the disease. Innate immunity is a critical step in the pathogenesis of OA, as injury or mechanical stress causes the release of damage-associated molecular patterns (DAMPs). These molecules then interact with pattern recognition receptors (PRRs), such as toll-like receptors (TLRs), which propagate inflammatory processes [113]. As described previously, cells taking part in RA pathogenesis can secrete or assimilate EVs to produce the desired cellular effects. In this section, we will focus on the role of EVs in the progression of OA.

To begin with, the cargo of exosomes derived from synovial fluid depends on the severity of OA. Exosomes isolated from the synovial fluid of patients with severe disease contain more cytokines and chemokines than those of patients with mild OA [114]. OA-EVs demonstrate a great abundance of molecules. Recently, Zhang et al. identified almost seven hundred peptides in EVs derived from synovial fluid, which correlate with severity scores. The majority of these peptides were associated with the immune system [115]. Consequently, these structures could be used in the diagnosis process. Similarly to RA, cells implicated in the pathogenesis of OA secrete EVs to induce particular responses in other cells. Thus, EVs take part in various processes, such as inflammation, cartilage degeneration or senescence.

### 4.2. Cartilage Degeneration and Inflammation

Cartilage damage and low-grade inflammatory responses are important mechanisms taking place in the pathogenesis of OA [112,116]. These responses can be induced through EVs. For instance, IL-1β-stimulated synovial fibroblast-derived exosomes induce OA-associated changes in articular chondrocytes. They decrease the expression of *COL2A1* and *ACAN*, key ECM markers, and promote *MMP-13*. Furthermore, exosomes from IL-1β-treated cells promote angiogenesis in human umbilical vein endothelial cells [117]. In addition, exosomes derived from OA-FLSs demonstrate the elevated expression of the lncRNA prostate cancer gene expression marker 1 (PCGEM1) versus vesicles from healthy FLSs. Exosomal lncRNA suppressed the proliferation of chondrocytes, promoted apoptosis and stimulated cartilage degradation. The mechanism of action of PCGEM1 involves the miR-142-5p/RUNX2 axis [118]. Similarly, the pathological environment can stimulate chondrocytes to secrete EVs that can enhance disease progression. For instance, Ni et al. demonstrated that EVs from IL-1β-pre-treated chondrocytes promoted inflammatory responses in LPS-primed macrophages through miR-449a-5p [119]. Similarly, the upregulation of circRNAs circ-BRWD1 and circ_0001846 in exosomes from IL-1β-stimulated chondrocytes were detected. Silencing these molecules inhibited inflammation and MMP-13 expression [120,121]. Circ-BRWD1 was found to act through sponging miR-1277 and promoting TRAF6. A previous study demonstrated that the expression of TRAF6 is elevated in damaged OA cartilage. Targeting TRAF6 with miRNA suppresses NF-κB and MAPK pathways [122]. In addition, exosomes derived from osteoclasts can also modulate the activity of chondrocytes. In surgery-induced OA mice models, these cells could transfer miRNA to chondrocytes to reduce their resistance to ECM damage and reduce the expression of tissue inhibitors of metalloproteinases (TIMPs) [123]. Furthermore, sclerotic OA subchondral bone osteoblasts secrete exosomes containing miR-210-5p, which are assimilated by articular chondrocytes. Cartilage cells transfected with miR-210-5p mimics show an elevated expression of hypertrophic markers, such as MMP-13 or ADAMTS5, as well as the decreased expression of chondrogenic factors, namely SOX9, COL2 or ACAN [124].

Liu and colleagues found elevated concentrations of circ-PRKCH in serum exosomes of patients with OA. Silencing circRNA reduced inflammatory IL-6 and TNFα. CircPRKCH positively regulated ADAMTS5 expression through the sponging of miR-502-5p. Exosomes extracted from IL-1β-stimulated chondrocytes promoted the expression of circPRKCH in other chondrocytes, which confirms the ability of articular cells to transfer this RNA molecule using EVs [125]. Similarly, another report demonstrated the upregulated lncRNA plasmacytoma variant translocation 1 (lncPVT1) expression in exosomes from the serum of OA patients compared to healthy controls. The suppression of lncRNA reduced the LPS-induced inflammatory responses in chondrocytes. LncPVT1 was found to exert its activity through the miR-93-5p/HMGB1 axis. HMGB1 is a member of DAMPs, which induces inflammation through NF-κB activation [126]. Taken together, these lines of evidence indicate that multiple RNA molecules have been found to mediate inflammation and cartilage degeneration in OA. Furthermore, these agents can be loaded and secreted in EVs to induce appropriate activities in other cells. Some vesicles can be detected in the blood, which could become beneficial in the diagnosis process.

### 4.3. Cartilage Calcification

OA pathogenesis is associated with mineralization disturbances and cartilage calcification [127]. Studies suggest that EVs can contribute to the calcification process. Liu et al. found that mimicking abnormal biomechanics present in the OA of temporomandibular joint (TMJ) promotes the development of calcified nodules and the expression of CD63 in primary condylar chondrocytes. The suppression of exosome formation decreased the number of calcified nodules. Importantly, in an in vivo experiment, the application of an exosome inhibitor suppressed cartilage calcification in the TMJs of rat models after a unilateral anterior crossbite stimulation [128]. Therefore, the major finding is that the suppression of exosome formation inhibited cartilage calcification. Thus, cells can use EVs to modulate cartilage impairment in different mechanisms. Interestingly, a disturbance of autophagy could promote the calcification associated with EVs. Autophagy involves recycling of organelles and intracellular molecules, which is important for energy homeostasis, and takes part in the response to stress or injury. It has been considered that autophagy plays a chondroprotective role and thus could suppress OA progression. Nevertheless, the associations between autophagy and apoptosis make the relationship with the disease more complex [129]. Recently, impaired autophagy flux has been associated with a secretion of LC3+ calcified EVs, structures that take part in cartilage calcification [130]. Since the inhibitors of autophagy stimulate the progression of OA [131], EVs could represent one of the potential pathogenetic mechanisms.

### 4.4. Cell Death Pathways

Promoting the viability of chondrocytes represents one of possible strategies investigated in the treatment of OA. The pathogenesis of the disease is associated with chondrocyte death. Various cellular death processes have been identified. For instance, ferroptosis is an iron-dependent mechanism that has been identified in the pathogenesis of OA [132]. Chondrocytes under inflammation or iron overload enter the process of ferroptosis. Moreover, the induction of ferroptosis in chondrocytes results in an elevated and decreased expression of MMP-13 and collagen type II, respectively [133]. Kong et al. showed that OA-FLS can promote ferroptosis and inflammation in chondrocytes by delivering miR-19b-3p [134]. Similarly, EVs have been found to take part in the process of pyroptosis, a form of programmed cell death which involves the activation of inflammasomes and caspases. This process can contribute to cartilage degeneration, synovial inflammation and pain [135]. Ebata et al. demonstrated that the stimulation of chondrocytes with EVs derived from LPS-treated macrophages resulted in the induction of inflammatory and catabolic markers. Importantly, these vesicles promoted the expression of pyroptosis-related molecules in chondrocytes, such as Nlrp3, Il18, Il1β and Gsmdm [136]. Thus, EVs can be secreted to induce death in other cells and then promote the progression of OA.

### 4.5. Senescence

Senescence is associated with morphological alterations, the accumulation of stress molecules and vacuolization. These alterations lead to the inhibition of growth and resistance to apoptosis, as well as to the production of senescence-associated secretory phenotypes (SASPs) which are inflammatory mediators and growth factors [137]. Culturing chondrocytes with a senescent cell-conditioned medium decreases the production of proteoglycan. The observed induction of senescence could result from the activity of EVs. Vesicles derived from senescent chondrocytes induced a similar phenotype in non-senescent cells. These results were found to occur through the transmission of miRNAs, as vesicles secreted by senescent cells contained less miR-140-3p and more miR-34a-5p than EVs from non-senescent cells [138]. Mir-34a-5p has been correlated with OA progression. Firstly, its expression is elevated in the tissues and plasma of patients compared to controls. Moreover, the treatment of human chondrocytes with mir-34a-5p mimics reduced the expression of COL2A1 and ACAN [139]. Therefore, OA chondrocytes can induce senescence in non-senescent cartilage cells through the transmission of miRNA, which decreases the production of ECM.

Connexins (Cx) form hemichannels and gap junctions to mediate cellular communication. Importantly, they can take part in the process of senescence induction. Firstly, the Cx43 expression is upregulated in OA, and its downregulation decreases the expression of pro-inflammatory mediators and MMPs [140]. Intriguingly, Cx43 can be expressed on the surface of EVs and form functional channels with targets cells to facilitate the release of cargo. The presence of Cx43 has been found on EVs derived from various cells, such as endothelial cells and retinal epithelial cells [141]. Valera-Eirin et al. found that small EVs derived from OA-patients were enriched in Cx43. The incubation of chondrocytes with EV-Cx43 lead to the downregulation of COL2A1 and ACAN, and promoted ERK signaling. Vesicles with Cx43 contained more proteins related to catabolic cell cycle processes, stress responses and the immune system. Importantly, EVs from OA-chondrocytes induced the expression of Cx43 and stimulated pro-inflammatory behaviors in bone and synovial cells. The authors observed that OA-chondrocytes can induce senescence in target cells through small EVs enriched with Cx43 [142]. Table 2 contains a summary of mechanisms involving EVs that take part in the OA pathogenesis.

## 5. The Role of Extracellular Vesicles in Suppressing Osteoarthritis

### 5.1. Mesenchymal Stem Cells and Osteoarthritis

As previously mentioned, MSCs possess immunomodulatory and regenerative features which have been explored in OA. The use of these cells has been examined clinically. A phase IIb clinical trial investigated the intra-articular treatment with the autologous AD-MSCs in OA patients. The use of MSCs was associated with significantly improved Western Ontario and McMaster Universities Osteoarthritis index score after 6 months of treatment [143]. Recently, positive effects of MSCs have been observed in a phase III trial [144].

### 5.2. Mesenchymal Stem-Cell-Derived Extracellular Vesicles and Chondrocytes

Dysregulated chondrocytes represent the major cells affected by and involved in OA. Therefore, suppressing the catabolic and senescent phenotype of chondrocytes could be of great interest. EVs derived from MSCs may represent an interesting strategy to achieve this goal. Stem-cell-derived EVs can promote chondrocyte proliferation and migration. Thus, these structures can suppress the negative effects induced by inflammatory conditions [145]. The cargo of MSCs-EVs includes immunomodulatory molecules that can inhibit chondrocyte senescence, such as lncRNA MEG3 [146]. A previous study demonstrated that MEG3 is downregulated in OA chondrocytes, whereas its forced expression stimulated ECM components through the miR-361-5p/FOXO1 pathway [147]. Furthermore, AD-MSC-EVs can enhance chondrogenesis by promoting the expression of chondrogenic markers that stimulate chondrocyte proliferation and differentiation [148]. Li et al. found that EVs secreted by adipose stem cells can exert their immunosuppressing role by transferring miR-338-3p, a molecule that targeted the runt-related transcription factor 2 (Runx2) [149]. Runx2 is considered as a significant molecule in the pathogenesis of OA, as its expression is elevated in OA mouse models. Furthermore, it has been associated with catabolic factors [150]. Nonetheless, its role in OA progression might be more complex. A recent study demonstrated that the heterozygous knockdown of Runx2 suppressed OA progression, but homozygous silencing promoted the disease. However, MMP-13 transcription was suppressed in both models [151]. Tofiño-Vian and colleagues also showed that EVs secreted by AD-MSCs suppressed the pro-inflammatory mediators stimulated by IL-1β in chondrocytes. Interestingly, the authors also observed that the expression of annexin 1 is elevated in EVs. The highest expression has been noted in microvesicles. The suppression of annexin 1 reduced the beneficial effects of microvesicles on the IL-6 and collagen II expression [152]. Importantly, vesicles secreted by MSCs suppress oxidative stress in chondrocytes. Guillen et al. demonstrated that microvesicles derived from AD-MSCs induced the expression of peroxiredoxin 6, an agent that can protect from oxidative injury and has been associated with the suppression of inflammation [153].

Interestingly, the pretreatment of synovial MSC-derived exosomes with LPS further improved the beneficial effects of EVs. LPS stimulation induced alterations in the expression patterns of miRNAs. In a study by Duan et al., 64 and 18 miRNA molecules were downregulated and upregulated, respectively. Among RNA molecules, let-7b was found to target ADAMTS5 and suppress cartilage degradation [154]. Similarly, EVs derived from BM-MSCs under hypoxic conditions also further improved the efficacy of these structures. Again, the altered environment significantly changed the expression of 29 miRNAs [155]. Furthermore, BM-MSCs cultured with curcumin produce EVs, which also improved the disease-suppressing properties of primary vesicles [156].

MSC-derived vesicles have been modified to include the bone morphogenic protein (BMP), which plays a role in cartilage repair and is considered chondroprotective. Importantly, recombinant BMPs are unstable, and studies have searched for an efficient delivery system [157]. The transfection of synovial MSCs with BMP-7 plasmid promoted the secretion of BMP-7-overexpressing exosomes. Importantly, synovial MSC-derived exosomes enhanced M2 macrophage polarization, which was further evident in the case of vesicles with BMP-7. In addition, the modified structures promoted chondrocyte proliferation and inhibited apoptosis. Importantly, MSC-exosomes and BMP-7-vesicles significantly reduced the histological score in vivo [158]. Another important protein in OA is matrilin-3 (MATN3), a member of the ECM adaptor family. Mutations in MATN3 have been associated with an increased risk of OA [159]. Synovial MSC-derived exosomes with MATN3 improved OA scores in mice. Long et al. identified that MATN3 interacted with IL-17A to prevent the activation of its downstream signaling (PI3K pathway) [160].

### 5.3. Extracellular Vesicles Derived from Other Cells

The above-mentioned evidence highlights the beneficial role of MSC-derived EVs. Nevertheless, EVs secreted by different cells have also been found to have immunomodulatory properties. For instance, platelet-derived exosomes demonstrated significant positive effects on chondrocytes, as well as in OA animal models [161]. Mir-126-3p has been found to suppress chondrocyte inflammation and induce proliferation. After the transfection of the miR-126-3p plasmid into synovial fibroblasts, this molecule was loaded and secreted in exosomes. Similarly to the direct chondrocyte transfection, the treatment with miR-125-3p containing exosomes reduced inflammation in chondrocytes. Moreover, the prepared exosomes were associated with clinical improvements in an in vivo experiment [162]. Furthermore, exosomes derived from FLSs transfected with lncRNA H19 can be absorbed by chondrocytes. Importantly, this lncRNA could diminish the negative effect of IL-1β on chondrocytes. Namely, the treatment with exosomes promoted COL2A1 and ACAN, as well as reduced the MMP-13 and ADAMTS5 expression. The molecule has been found to act through the miR-106b-5p/TIMP2 axis [163]. As previously mentioned, TIMP2 takes part in the resistance to extracellular matrix damage. Importantly, reduction in its levels has been correlated with OA in mice [164]. Moreover, H19 has been found in exosomes secreted by UC-MSCs to modulate pain reactions [165], which demonstrated the broad interaction networks of ncRNA and their contribution to various cellular processes.

Importantly, Wang and colleagues point to the importance of a healthy synovium in OA. A co-culture of EVs derived from healthy FLSs with chondrocytes resulted in the enhanced expression of ECM markers, together with reduced pro-inflammatory cytokines. These effects could be mediated by miR-150-3p which inhibited the Trim14/NF-κB/IFNβ pathway [166]. These observations seem to be confirmed by a recent study, in which the authors treated OA mice with exosomes derived from FLSs extracted from neonatal mice. Clinical arthritis scores declined after the treatment. Moreover, the vesicles could carry miR-25-3p to inhibit pyroptosis [167]. Additionally, the downregulation of SOX9 has been found in OA tissues. Promoting the expression of this transcription factor reduces the production of TNF-α and inhibits apoptosis in IL-1β-treated chondrocytes. Moreover, introducing the SOX9 lentivirus vector into the surgically induced OA animal models improved clinical scores (Osteoarthritis Research Society International and the synovitis scores) [168]. SOX9 mediates chondrogenesis and regulates the expression of ECM molecules [169]. Intriguingly, SOX9 could be transported from M2 macrophages to chondrocytes through exosomes, which could support the functions of the latter cells [170].

### 5.4. Extracellular Vesicles Containing Mitochondria

Impaired mitochondria have been suggested to play a role in OA progression. Various pathologies of these organelles have been observed, which could contribute to the disease progression. These disruptions include impairment of the mitochondrial respiratory chain, excessive generation of reactive oxygen species (ROS) and mitochondrial DNA damage, among others [171]. Importantly, EVs have been found to contain mitochondrial proteins, mitochondrial DNA or even functioning organelles, and their role have been investigated in a number of diseases [172]. Intriguingly, vesicles containing mitochondria can be assimilated by chondrocytes [173]. Yu et al. showed that BM-MSC-derived EVs could stimulate mitochondrial function in chondrocytes. The authors showed that microvesicles contained mitochondria, and these structures could ameliorate the progression of OA. Moreover, beneficial effects were observed when OA models were treated with BM-MSC-derived mitochondria [174]. Overall, the delivery of functioning mitochondria to chondrocytes is an interesting approach that seems to induce beneficial effects and should be further explored.

### 5.5. Extracellular Vesicles and Osteoarthritis Pain

Pain is the hallmark symptom of OA. Inflammation and structural impairment of the elements of the joint contribute to pain development. Multiple ion channels and neuropeptides have been found to take part in the transduction of nociception. Furthermore, neuropathic mechanisms are also considered to play a role in OA pain processes [175]. Studies have demonstrated that EVs can suppress pain-related signaling.

Nerve growth factor (NGF) is a significant mediator of pain transduction that can be secreted by various cells involved in OA pathogenesis. Surprisingly, an in vivo study showed that silencing NGF provided pain relief but seemed to promote disease progression [176]. Interestingly, MSC-derived EVs have been found to suppress excitability in the dorsal root ganglion (DRG) neuron stimulated with NGF, which could be associated with the inhibition of pain reactions in mice [177].

**Figure 2:**
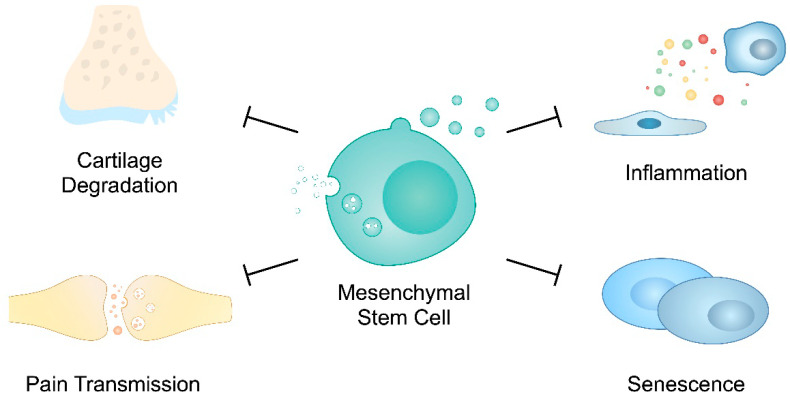
Mesenchymal stem-cell-derived extracellular vesicles have been found to suppress cartilage degradation, inflammation and senescence, as well as pain transmission, which could be used to suppress osteoarthritis progression.

Moreover, studies have demonstrated that the calcitonin gene-related peptide (CGRP) takes part in pain transduction in OA. Its expression is elevated in OA patients and has been correlated with somatic pain intensity [178]. He et al. demonstrated that BM-MSC-derived exosomes reduced CGRP levels in rat OA DRG tissue [145]. Furthermore, Lu and colleagues have developed exosomes loaded with miR-204. Intra-articular injections of those structures provided pain relief in animal OA models. The authors identified that miR-204 could achieve pain suppression through inhibition of the SP1- LDL receptor-related protein 1 (LRP1) axis [179]. Overall, MSC-derived EVs have been demonstrated to positively regulate pain responses in OA. Figure 2 summarizes major positive roles of EVs secreted by MSCs in OA progression.

### 5.6. Boosting the Efficacy of Extracellular Vesicles

Recently, a trend towards precision therapy in medicine can be observed. Importantly, EVs can be modified to precisely deliver cargo that can suppress the disease. For instance, Liu and colleagues identified miR-223 as a potential agent in UC-MSCs-EVs which suppresses the NLRP3 inflammasome. Subsequently, EVs were engineered to include the collagen II binding peptide and overexpress miR-223. These structures amplified the beneficial effects of UC-MSCs-EVs. Thus, these structures contained a precise therapeutic agent and were equipped with proteins which allowed for precise targeting [180]. Moreover, a modification of exosomes to include the chondrocyte-affinity peptide has recently been found to further improve the efficacy of vesicles derived from subcutaneous fat MSCs loaded with miR-199a-3p [181]. In addition, EVs can be modified to alter their surface charge, which can improve their intra-articular bioavailability [182].

### 5.7. Apoptotic Bodies

Apoptosis is a programmed cell death mechanism during which cells release large ABs. Interestingly, these structures can be functional and modulate the number of cellular processes [183]. Importantly, macrophage reprogramming can involve ABs. The introduction of M2-derived ABs significantly protected bone loss in the OA mice model. M2-ABs have been found to reprogram M1 macrophages into the M2 subtype, which was associated with a reduced expression of pro-inflammatory cytokines. These effects were found to occur through the presence of miR-21-5p [184]. Furthermore, ABs from MSCs have been found to induce the M2 macrophage, which has been investigated in the context of wound healing [185]. Such MSC-derived structures can influence macrophage polarization through transporting miR-21-5p as well [186]. Moreover, studies demonstrated the beneficial properties of MSC-derived ABs in various models and diseases [187].

## 6. Conclusions

Overall, the aim of this review was to describe the broad roles of EVs in the pathogenesis and treatment of RA and OA. Cells implicated in the progression of these diseases can communicate through EVs and induce various responses. In RA, these vesicles can regulate autoimmunity and inflammed bone impairment, as well as T cell differentiation. Nevertheless, these vesicles can also carry immunomodulatory cargo, which suppresses inflammation and the proliferation of RA-FLSs. Similarly, EVs have been found to act in both ways to promote or suppress OA. Cells can modulate each other’s activities by transporting ncRNA molecules, which mediate gene expression. Interestingly, these RNA molecules form a broad machinery of molecular interactions, which can impact various signaling pathways. EVs can be modified to overexpress a required molecule or contain a binding protein to improve bioavailability. The use of EVs can be an interesting continuation of ncRNA studies, as molecules downregulated in the RA/OA tissues could be loaded into exosomes and delivered to the affected joint. Furthermore, monitoring the content of EVs might help in predicting the treatment response [188]. Current evidence suggests that MSCs and MSC-derived EVs play an enormous role in cartilage regeneration and the suppression of inflammation. Future studies should investigate the potential efficacy and safety of this cell-free therapy. Importantly, EVs can also be used as carriers for already known medicines. For instance, encapsulating dexamethasone in macrophage-derived and modified exosomes showed significant clinical activity in the RA mice model [189]. MSC-derived EVs are being examined as therapeutic agents in other diseases [190,191].

## Figures and Tables

**Table 1:** Summary of the processes involved in the pathogenesis of rheumatoid arthritis mediated by extracellular vesicles.

Mechanism Involved in the Pathogenesis of RA	Role of EVs in RA Progression	Reference
Autoimmunity	EVs are associated with the presence of citrullinated proteins	[21,22,26,27,28]
	Immune complexes formed with EVs induce pro-inflammatory responses	
Inflammation	EVs contain TNF-α which can stimulate NF-κB pathway	[33,34,37,38,42]
	Inflammatory environment stimulates the secretion of EVs with ncRNA that further modulate inflammation	
	Microparticles derived from synovial fluid promote inflammatory responses in FLSs	
	RA-FLS-derived exosomes promote macrophage migration through pentraxin 3	
	Exosomes derived from synovial fibroblasts containing miR-424 promote Th17 T cell differentiation	
Bone degradation	Exosomes derived from RA-FLSs suppress osteoblast differentiation	[47,48,55]
	TNF-stimulated FLS contain miR-221-3p which inhibits osteoblast differentiation	
	Th17 cells stimulated with cigarette smoke-enriched medium or aryl hydrocarbon receptor agonist promote the secretion of EVs containing miR-132 that stimulates osteoclastogenesis	
Extracellular matrix degradation	RA-FLSs secrete microvesicles with proteases that degrade aggrecan	[56]
Angiogenesis	RA-FLSs secrete exosomes containing inhibitor of DNA binding 1, which has pro-angiogenic features	[58,60]
	RA-FLS-derived EVs can promote angiogenesis by secreting miR-1972 that regulates p53/mTOR	

**Table 2:** Summary of the processes involved in the pathogenesis of osteoarthritis mediated by extracellular vesicles.

Mechanism Involved in the Pathogenesis of OA	Role of EVs in OA Progression	Reference
Inflammation	Exosomes derived from IL-1β-stimulated synovial fibroblasts promoted *TNFα* expression in chondrocytes.	[117,119,120,126]
	EVs derived from IL-1β-stimulated chondrocytes further promoted IL-1β production in macrophages.	
	Silencing circ-BRWD1, which can be secreted in exosomes by chondrocytes, reduced the expression of IL-6 and IL-8.	
	Chondrocyte stimulation with LPS promotes the secretion of exosomal lncRNA PVT1, which plays a role in inflammatory responses.	
Cartilage Degradation	Exosomes derived from IL-1β-stimulated synovial fibroblasts promoted *MMP-13* and suppressed *ACAN* expression.	[117,118,120,121,123,125,126]
	LncRNA PCGEM1 present in exosomes derived from OA-FLSs promoted chondrocyte apoptosis. Furthermore, it promoted MMP-13 and inhibited COL2A1 and Aggrecan expression in chondrocytes.	
	IL-1β-stimulated chondrocytes secrete exosomes with elevated circ-BRWD1 and circ_0001846 expression, which take part in cartilage degradation.	
	Osteoclast secrete exosomes containing miRNA that can suppress tissue inhibitors of metalloproteinase (TIMPs) in chondrocytes.	
	IL-1β-stimulated chondrocytes can transfer circ-PRKCH in exosomes, which can modulate ADAMTS5 expression.	
	Chondrocyte stimulation with LPS promotes the secretion of exosomal lncRNA PVT1, PVT1 knockdown reduced MMP-13 and promoted aggrecan.	
Cartilage Calcification	EVs mediate the process of cartilage calcification, observed in the pathogenesis of OA.	[128,130]
Cell death	Exosomes derived from OA-FLSs promote ferroptosis in IL-1β-stimulated chondrocytes.	[134,136]
	EVs derived from LPS-stimulated macrophages promote pyroptosis in chondrocytes.	
Senescence	EVs derived from senescent cells induce senescence in chondrocytes.	[138,142]
	EVs enriched with Cx43 promote senescent state in other cells.	

## Data Availability

Not applicable.
